# Phytohormones (Auxin, Gibberellin) and ACC Deaminase In Vitro Synthesized by the Mycoparasitic *Trichoderma* DEMTkZ3A0 Strain and Changes in the Level of Auxin and Plant Resistance Markers in Wheat Seedlings Inoculated with this Strain Conidia

**DOI:** 10.3390/ijms20194923

**Published:** 2019-10-04

**Authors:** Jolanta Jaroszuk-Ściseł, Renata Tyśkiewicz, Artur Nowak, Ewa Ozimek, Małgorzata Majewska, Agnieszka Hanaka, Katarzyna Tyśkiewicz, Anna Pawlik, Grzegorz Janusz

**Affiliations:** 1 Department of Environmental Microbiology, Maria Curie-Sklodowska University, Akademicka St. 19, 20-033 Lublin, Poland; jolanta.jaroszuk-scisel@poczta.umcs.lublin.pl (J.J.-Ś.); artur.nowak@poczta.umcs.lublin.pl (A.N.); majewska@poczta.umcs.lublin.pl (M.M.); 2 Military Institute of Hygiene and Epidemiology, Lubelska St. 2, 24-100 Puławy, Poland; 3 Department of Plant Physiology, Maria Curie-Sklodowska University, Akademicka St. 19, 20-033 Lublin, Poland; agnieszka.hanaka@poczta.umcs.lublin.pl; 4 ŁUKASIEWICZ Research Network—New Chemical Syntheses Institute, Tysiąclecia Państwa Polskiego Ave. 13a, 24-110 Puławy, Poland; katarzyna.tyskiewicz@ins.pulawy.pl; 5 Department of Biochemistry, Maria Curie-Sklodowska University, Akademicka St. 19, 20-033 Lublin, Poland; anna.pawlik@poczta.umcs.lublin.pl (A.P.); gjanusz@poczta.umcs.lublin.pl (G.J.)

**Keywords:** *Trichoderma*, auxin indoleacetic acid (IAA), gibberellins, 1-aminocyclopropane-1-carboxylic acid (ACC) deaminase, plant resistance markers, mycoparasitism

## Abstract

Both hormonal balance and plant growth may be shaped by microorganisms synthesizing phytohormones, regulating its synthesis in the plant and inducing plant resistance by releasing elicitors from cell walls (CW) by degrading enzymes (CWDE). It was shown that the *Trichoderma* DEMTkZ3A0 strain, isolated from a healthy rye rhizosphere, colonized the rhizoplane of wheat seedlings and root border cells (RBC) and caused approximately 40% increase of stem weight. The strain inhibited (in over 90%) the growth of polyphagous *Fusarium* spp. (*F.* *culmorum*, *F.* *oxysporum*, *F.* *graminearum*) phytopathogens through a mechanism of mycoparasitism. Chitinolytic and glucanolytic activity, strongly stimulated by CW of *F.* *culmorum* in the DEMTkZ3A0 liquid culture, is most likely responsible for the lysis of hyphae and macroconidia of phytopathogenic *Fusarium* spp. as well as the release of plant resistance elicitors. In DEMTkZ3A0 inoculated plants, an increase in the activity of the six tested plant resistance markers and a decrease in the concentration of indoleacetic acid (IAA) auxin were noted. IAA and gibberellic acid (GA) but also the 1-aminocyclopropane-1-carboxylic acid (ACC) deaminase (ACCD) enzyme regulating ethylene production by plant were synthesized by DEMTkZ3A0 in the liquid culture. IAA synthesis was dependent on tryptophan and negatively correlated with temperature, whereas GA synthesis was positively correlated with the biomass and temperature.

## 1. Introduction

The hormones produced by plants affect not only their growth and development, but also the interaction between microorganisms and higher organisms (plants and animals), mainly by the determination of the microbiome of plant tissues and animal intestines structure [1,2]. Such interaction is based on plant-colonizing microorganisms (bacteria and fungi) synthesizing their own phytohormones, affecting the hormonal balance of the plant and modifying the interaction with microorganisms [3,4]. Phytohormones play an important role in agriculture and medicine, which is reflected in the interest in the industrial production of phytohormones, especially using fungal cultures [1]. These organic substances are able to modify the plant physiological functions and growth, even at low concentrations. Phytohormones are divided into five main categories: Auxins, cytokinins, ethylene, gibberellins (GAs), and abscisic acid (ABA) but a number of other new phytohormones or hormone-like substances such as brassinosteroid, oligosaccharines, bioamines, salicylates–salicylic acid (SA), and jasmonic acid (JA) have been described [5]. A low level of indoleacetic acid (IAA), i.e., the main representative of the auxin group of phytohormones, stimulates root elongation, while a high level influences the formation of lateral and adventitious roots. GAs control processes of plant development, including seed germination, stem extension, flowering, and aging. Phytohormones and hormone-like compounds can provide successful seed germination or normal growth of plants by a regulation of a symbiotic relationship between plants and, for instance, mycorrhizal fungi or nodule bacteria [3,5].

Among the most commonly occurring fungi isolated from different environments, dead wood, bark, other fungi, building materials, and animals, including humans, are species of the mycotrophic filamentous ascomyceteous genus *Trichoderma* (Hypocreales, Hypocreaceae; teleomorph Hypocrea). They are characterized by a high opportunistic potential and adaptability to ecological conditions [6]. The main reservoirs for *Trichoderma* communities are soil, rhizosphere, and plant microbiomes [6,7,8].

The taxonomy of the genus *Trichoderma* is complicated and belongs to the most dynamically developing branches of mycology [9,10]. *Trichoderma* or *Hypocrea* could have been adopted as the genus name but with priority to asexual *Trichoderma* over Hypocrea *(*with sexually typified type species), *Trichoderma* was given [11]. The *Trichoderma* taxonomy has gone through a remarkable transformation since 1969, when Rifai concluded that the genus includes more than a few species and divided the studied strains into nine “aggregate” species, including *T. koningii* [10]. Three independent, distinct lineages of *T. koningii* were separated based on phylogenetic analyzes (partial sequences of the translation-elongation factor 1 alpha, and fragments of actin and calmodulin genes) and the first of them, alongside *T. koningii* and *T. ovalisporum*, seven new species were included, among them *T. koningiopsis*. *T. koningiopsis* is mentioned on the list of 252 accepted *Trichoderma* species names [11].

Among the *Trichoderma* spp., strains with a negative effect on phytopathogens and a positive effect on plant growth as well as the ability to colonize the rhizosphere and plant tissue (endophytes) are particularly sought [12,13,14].

*Trichoderma* species use a wide array of biological control mechanisms such as antibiosis, antagonism, mycoparasitism, and induction of plant defense responses [15]. There are well-known mycoparasites of several fungi, able to secrete cell wall degrading enzymes (CWDE), such as chitinases and glucanases, to aid in parasitism [16,17]. *Trichoderma* species are well known for the production of bioactive metabolites that play an important role in the mycoparasitic or entomopathogenic lifestyles of the fungus and in auto-regulatory processes in mycoparasitism/competition and interaction with the plant, e.g., the induction of resistance in plant hosts [18]. The *Trichoderma* species attach to the hyphae of the fungal or oomycete host, creates a coil around the host hyphae, forms structures similar to hooks, appressoria, or papillae, and penetrates the cell wall (CW) by the production of CWDEs [6,19,20]. Interestingly, the *Trichoderma* spp. colonizes the roots of plants in a characteristic way similar to the interaction with the fungal host, where hyphae grow alongside on roots and root hairs [21].

*Fusarium* is a widely dispersed soil-borne filamentous phytopathogenic fungus belonging to Division: Ascomycota, Class: Sordariomycetes, Order: Hypocreales, and Family: Nectriaceae. It causes serious damage to a number of field, cereal, ornamental, forest, and horticultural crops in different climatic conditions [22,23,24,25,26,27,28]. Fungi belonging to the *Fusarium* genus are among the top ten of the greatest economically and scientifically important phytopathogens. For instance, *F. graminearum* (predominantly infecting cereal plants) and *F. oxysporum* (having a wide host range and mainly infecting non-cereal species) take the fourth and fifth place on the list, respectively [29]. Acting in multi-species complexes, *F. culmorum* usually together with *F. avenaceum*, cause a wide variety of cereal diseases, including crown rot (foot rot), seedling blight, dryland root rot, foot rot (dryland foot rot), and scab (*Fusarium* head blight—FHB), mainly in the wheat. The hexaploid wheat species usually called “common” or “bread” *Triticum aestivum* grown throughout the world is the third (after other major cereals rice and maize) most important crop in terms of global production and has major importance in a human diet and economy [30]. The *Fusarium* spp. also causes rot on apples [31], sugarcane wilt or pokkahboeng [24,27], bakanae in rice [23], oil palm wilt [25], and panama disease [28] and can produce mycotoxins such as trichothecenes and fumonisins, which have a great impact on the health of humans and livestock [32].

It should be emphasized that so far it has not been possible to develop a fully effective, environmentally safe, and economically viable biological method of protecting plants against the *Fusarium* spp. However, a strong biocontrol potential of many strains of plant growth promoting the rhizobacteria (PGPR) species has been demonstrated. Examples of PGPR are the species from the *Bacillus*, *Burkholderia*, *Pseudomonas* genera [33,34,35], yeasts belonging to the genera *Cryptococcus*, *Rhodotorula*, and *Saccharomyces* [36], and nonpathogenic endophytic *Fusarium* spp. strains, e.g., *F. oxysporum* [37] and *F. culmorum* [38]. Recently, hypovirulent isolates infected by mycoviruses are of great interest as biocontrol agents [26].

Over 60% of registered biopesticides have been developed with the use of *Trichoderma*, but only a few of them are intended to protect plants against fusariosis [39,40]. Most of these biopesticides contain *Trichoderma* strains capable of inducing resistance to plant pathogens, i.e., inducing an indirect biocontrol mechanism mediated by plants in which the biocontrol agent and the phytopathogen do not have to come into direct physical contact [41,42,43,44,45]. Taxonomically, diverse plants (monocots and dicots) react to the presence of a pathogen with a rapid expression of defense-related genes [46]**.** The colonization of plants by nonpathogenic fungi and bacteria, especially by endophytes, cause plants to enter a ‘‘primed state’’ and can lead to a broad-spectrum resistance to pathogens and insects [47,48,49]. Among endophytes, i.e., microorganisms present in plant tissues without causing any apparent symptoms [50], fungal endophytes are distinguished by their widespread, universality, and diversity [51].

It was shown that *Trichoderma* strains can induce systemic resistance not only after soil or root inoculation but also after the introduction of conidia on the leaves [52,53].

Elicitors (resistance inducers) are active in the form of monomers and can be divided into three broad categories: Proteins with enzymatic activity, avirulence-like gene products, and low molecular weight compounds released from CWs (either fungal or plant) as a result of the activity of hydrolytic enzymes (e.g., chitinase, glucanase) [45]. The elicitors affecting plants at various stages of development are extremely diverse and mainly include derivatives of microbial polymers (produced by bacteria, fungi, and oomycota) composed of sugars, fats (lipopolysaccharides), amino acids (elicitins; i.e., chitin), glucans, fungal ergosterol, and flagellin, cold shock proteins; transglutaminase, and enzymatic proteins (invertase in yeast and xylanase in *Trichoderma*) [54,55].

Microbial strains synthesizing a number of CWDEs can cause the formation of exoelicitors—products of own CW degradation and endoelicitors—products of the plant CW degradation. Furthermore, CWDEs allow these microorganisms not only to enter the target pathogen but also to utilize their CW components (β, α-1,3 glucan, and chitin) as nutrients [56,57]. The plants detect the composition of elicitors and respond by the induction of host resistance pathways [58]. The phenyloalanine ammonia lyase (PAL) is a crucial enzyme in the phenylpropanoid biosynthetic pathway, which is active in a resistance of several plant species against pathogens and plays an important role in the production of phytoalexins (flavonoids) and lignin biosynthesis [59,60]. Flavonoids have the ability to modulate the ROS-signaling cascade and strongly affect phytohormone (especially auxin) catabolism, transport, and signaling [61]. The induction of systemic resistance via the JA/ethylene signaling pathway was reported primarily for PGPR; however, it is also operative for many mycorrhizal [62] and biocontrol fungi [41,45].

The plants do not have adaptive immunity but they activate two forms of plant innate immunity (immune signaling pathways): (1) Pathogen-associated molecular pattern (PAMP)-triggered immunity (PTI) and (2) effector-triggered immunity (ETI), i.e., R-gene-based immunity, previously known as horizontal and vertical resistance, respectively [63,64,65,66,67]. The induction of both these immune pathways results in evoking systemic acquired resistance (SAR) [68]. In PTI, microbe-associated molecular patterns (MAMPs) and damage-associated molecular patterns (DAMPs), mainly including CW or extracellular protein fragments, peptides, nucleotides, and amino acids, induce corresponding pattern recognition plasma membrane-localized receptors (PRRs) of the host plant [64,69].

Microbes are able to synthesize and secrete phytohormones influencing two classes of hormones, i.e., SA and JA, which are the most important for plant immune responses to pathogens [70,71]. SA and JA with reactive oxygen species (ROS) and ethylene are involved in the network of signal transduction and the accumulation of pathogenesis-related (PR) proteins [72,73]. PR proteins that show an enzymatic activity such as glucanase (PR2) and chitinases (PR3) or peroxidase (PR9), are grouped into 17 families based on their sequence as well as enzymatic and other biological activities [73,74,75].

The mechanisms that mediate hormonal crosstalk are the object of much research due to their complicatedness and lack of explanation. DELLA proteins play an important role in a hormonal crosstalk, positively regulating the JA pathway as negative regulators of GA signaling [71,76,77].

The activity of the ACC (1-aminocyclopropane-1-carboxylic acid) deaminase enzyme limits the level of ethylene in the plant by converting the ACC direct precursor to this hormone, which is greatly important in promoting plant growth [78,79,80,81].

The maintenance of the effectiveness of biological protection preparations during periods of reduced temperature requires testing the activity of ingredients of the agents and appropriate selection of strains [82]. The optimum temperature for growth differs among the *Trichoderma* species [83,84], but most *Trichoderma* spp. strains are mesophilic with the greatest saprophytic activity from 15 to 21 °C [40,85]. Various mechanisms of *Trichoderma* strains action have already been reported [6,15,16,17,20,28,41] but the literature on strains combining mechanisms, such as mycoparasitism, phytohormone and ACC deaminase synthesis, root colonization and induction of plant resistance and at the same time about the interrelationship of these mechanisms, is still very limited.

The aim of this study was to prove changes in plant tissues after inoculation with the *Trichoderma* strain with biostimulatory and biocontrol potential. We focus on (I) the in vitro ability of this strain to produce phytohormones (IAA and GA) and the enzyme regulating the level of plant ethylene (ACC deaminase); (II) the mycoparasitic interaction of phytopathogens from the genus *Fusarium* with the potential for generating resistance elicitors from fungal CW, and (III) in vivo root colonization, stimulation of plant growth, induction of plant resistance, and influence of IAA levels on the plant. The tested plant host (monocotyledonous cereal—wheat) and phytopathogenic *Fusarium* spp. strains (*F. graminearum*, *F. culmorum, F. oxysporum*) appear to be a good model system for comparative studies of *Trichoderma*-plant host interactions.

## 2. Results

### 2.1. Determination of Phylogenetic Position of DEMTkZ3A0 Isolate

**Figure 1:**
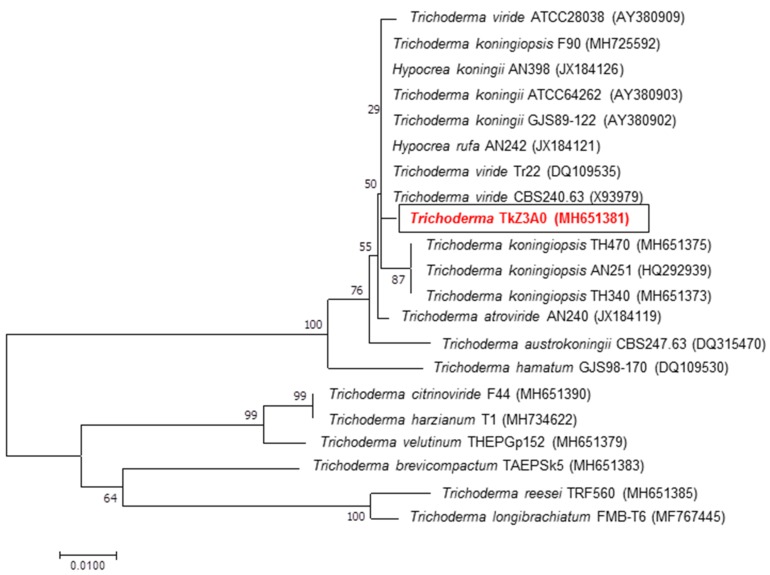
The evolutionary relationships of *Trichoderma/Hypocrea*. The evolutionary history was inferred using the Neighbor-Joining method [86]. The optimal tree with the sum of branch length = 0.26511463 is shown. The percentage of replicate trees in which the associated taxa clustered together in the bootstrap test (500 replicates) are shown next to the branches [87]. The tree is drawn to scale, with branch lengths in the same units as those of the evolutionary distances used to infer the phylogenetic tree. The evolutionary distances were computed using the Jukes–Cantor method [88] and are in the units of the number of base substitutions per site. The analysis involved 21 nucleotide sequences. All positions containing gaps and missing data were eliminated. There was a total of 379 positions in the final dataset. Evolutionary analyses were conducted in MEGA7 [89].

In order to determine the phylogenetic position of the tested fungal isolate, a 600 bp fragment of the ITS region was obtained from PCR with ITS1 and ITS4 primers, followed by direct sequencing. The sequence of this product revealed over 99% identity of DEMTkZ3A0 to *Trichoderma koningiopsis*, as shown in the NCBI-BLAST search system. The following GenBank accession number was assigned to the nucleotide sequence determined in this study: MH651381. The NJ (Neighbor Joining) method was employed to construct a phylogenetic tree for DEMTkZ3A0 and other strains described in databases (Figure 1). The fungus was clustered together with other closely related *Trichoderma* strains belonging to a very well-defined cluster (*Rufa* clade), which was further supported with a bootstrap value (100% bootstrap).

### 2.2. Growth Rate of Trichoderma DEMTkZ3A0 Strain Under Various Temperature and Carbon Conditions

**Figure 2:**
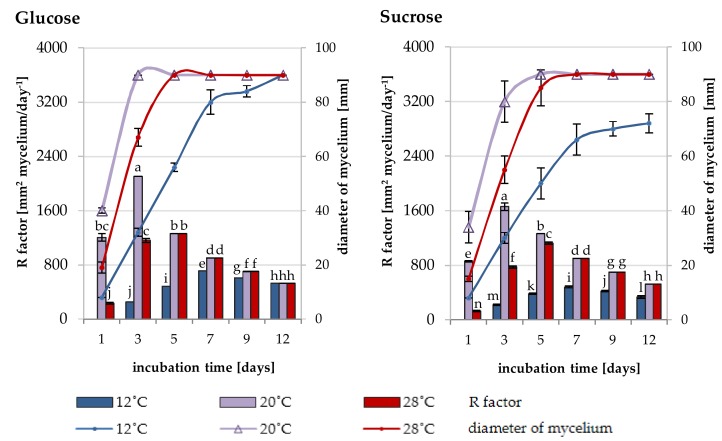
The growth of *Trichoderma* DEMTkZ3A0 strain on solid mineral Reyes and Byrde (RB) [90] medium supplemented with 1% glucose or 1% sucrose expressed as colony diameter and the growth rate (factor R) changes during 132 day incubation at three temperature 12, 20, and 28 °C.

The significant differences in the increase of *Trichoderma* DEMTkZ3A0 strain growth on solid medium were observed (Figure 2). The dynamics of growth expressed by the increase in the DEMTkZ3A0 colony diameter and the growth rate (factor R) largely depended on the type of sugar used (glucose or sucrose) and the temperature (12, 20, and 28 °C). On the glucose medium, the value of R factor increased until the 3rd day of incubation at 20 °C and up to the 5th day at 28 °C. In turn, R factor increased up to the 5th day at 20 °C and to the 7th day at 28 °C on the sucrose medium. In the following days of incubation, the R value was constant. The growth of the tested strain was different at the low temperature (12 °C), in which a strong increase in the R value was observed until the 7th day of incubation, followed by an upward trend until the 12th day of incubation. On the latter day, the R value in the glucose-supplemented culture was identical for the three temperature values. In turn, this parameter (R value) was 4-times lower than at 20 and 28 °C in the sucrose-enriched cultures.

### 2.3. Mycoparasitic Effect of Trichoderma DEMTkZ3A0 Strain on Phytopathogens

**Figure 3:**
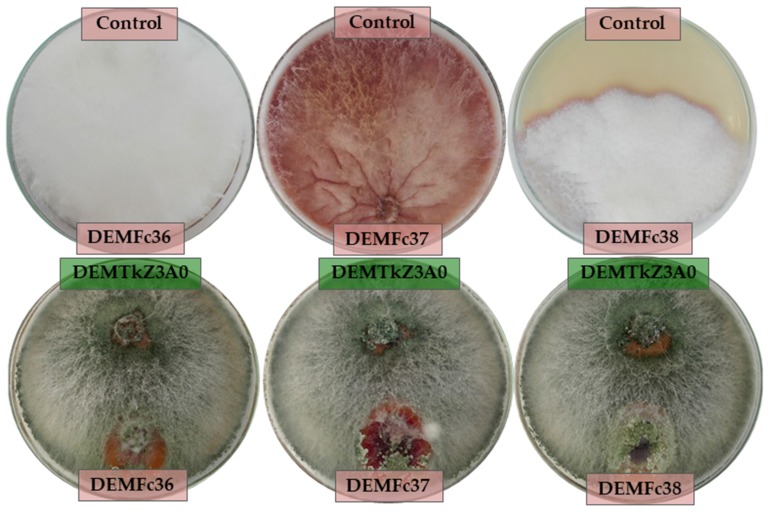
The macrographs of positive biotic effect of *Trichoderma* DEMTkZ3A0 strain in the interaction with phytopathogenic *Fusarium* spp. strains: *F. graminearum* DEMFc36, *F. culmorum* DEMFc37, and *F. oxysporum* DEMFc38 in comparison to the control on PDA medium, after 14 days of incubation at 20 °C.

**Figure 4:**
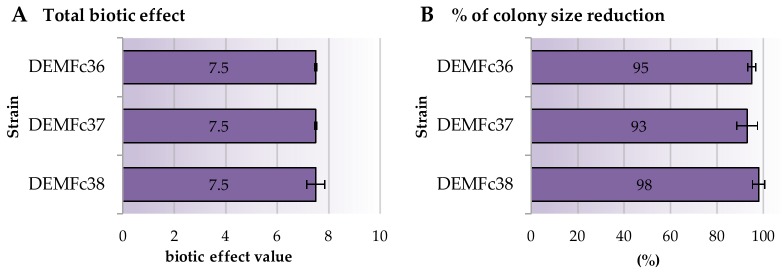
The values of total biotic effect (**A**) of the *Trichoderma* DEMTkZ3A0 strain and (**B**) reduction of colony size (%) of pathogenic *Fusarium* spp. strains (*F. graminearum* DEMFc36, *F. culmorum* DEMFc37, and *F. oxysporum* DEMFc38) by the DEMTkZ3A0 strain on potato dextrose agar (PDA) medium, after 14 days of incubation at 20 °C.

In this study, the ability of the DEMTkZ3A0 strain to limit and inhibit the growth of pathogenic *Fusarium graminearum* DEMFc36, *Fusarium culmorum* DEMFc37, and *Fusarium oxysporum* DEMFc38 strains on potato dextrose agar (PDA) medium after 14 days of incubation at 20 °C was determined. The ability of DEMTkZ3A0 strain to enter into a mycoparasitic interaction with the studied phytopathogens was also examined (Figure 3, Figure 4A,B).

The DEMTkZ3A0 strain clearly restricted and inhibited the growth of DEMFc36, DEMFc37, and DEMFc38 phytopathogens, achieving a positive biotic effect (Figure 3). The tested DEMTkZ3A0 strain showed the ability to surround the colonies of phytopathogens and reduce colony size, obtaining a total biotic effect of +7.5 in interaction with the *Fusarium* pathogens (Figure 4A). The DEMTkZ3A0 strain reduced the colony of pathogenic *Fusarium* strains by almost 100% compared to the control (Figure 4B).

**Figure 5:**
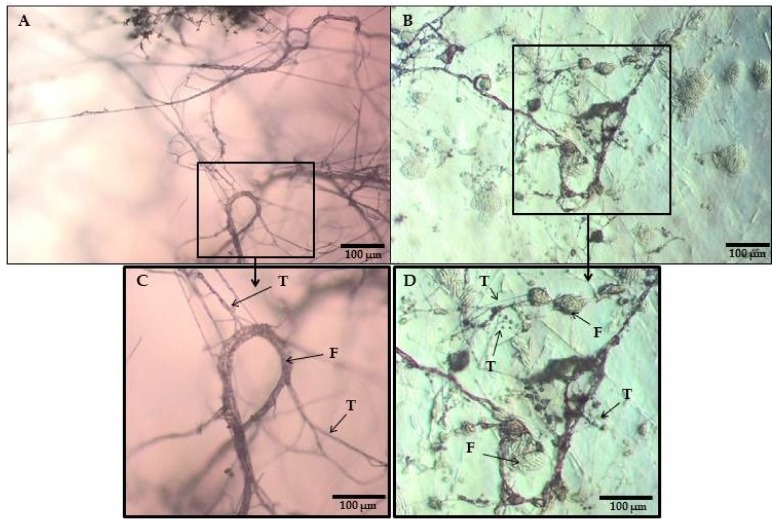
The microscopic (light microscope—LM) evaluation of the interaction of the *Trichoderma* DEMTkZ3A0 strain on phytopathogens: (**A**,**C**) *F. oxysporum* DEMFc38 and (**B**,**D**) *F. culmorum* DEMFc37. The DEMTkZ3A0 strain showed a characteristic picture of the parasitic interaction with both strains: Adhesion, chemotaxis towards pathogen’s hyphae (C), and coiling around the pathogen spores (D). Abbreviations: F—*Fusarium* spp. hyphae or spores; T—DEMTkZ3A0 strain hyphae.

The microscopic analysis showed that the hyphae of *Trichoderma* DEMTkZ3A0 strain interacted with the pathogens by chemotaxis and adhesion and formed a characteristic image of the parasitic interaction in relation to the hyphae of phytopathogenic strains, including the formation of appressorium-like structures and coiling around the hyphae (Figure 5A,C) and spores (phialides with macroconidia) (Figure 5A,C) of *Fusarium* spp. phytopathogens, causing the lysis of pathogens CW (Figure 5A–D).

### 2.4. Chitinolytic and Glucanolytic Activities in Culture Supernatants of Trichoderma Strain Grown in Media Containing Glucose, Chitin and Lyophilized Cell Wall of Phytopathogenic F. culmorum Strain

**Figure 6:**
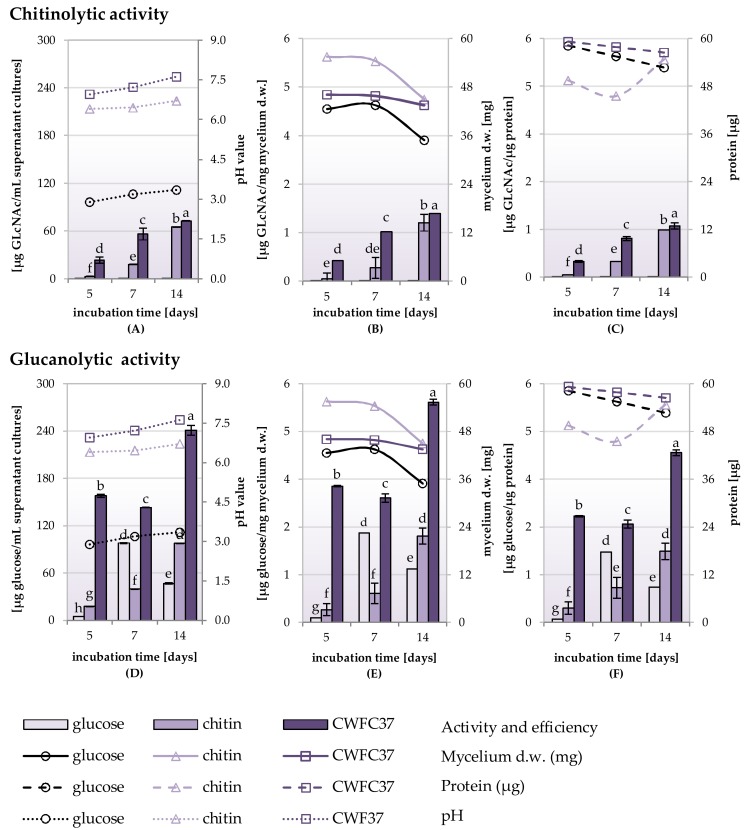
The chitinolytic (**A**,**B**,**C**) and glucanolytic activity (**D**,**E**,**F**) of the *Trichoderma* DEMTkZ3A0 strain on three carbon sources: Glucose, chitin, and lyophilized cell wall of the pathogenic *F. culmorum* strain (CWFC37) after 5, 7, and 14 days of incubation based on released *N*-acetyl-d-glucosamine (μg GLcNAc/mL (**A**) or μg GLcNAc/mg mycelium (**B**) or GLcNAc /μg protein (**C**) for chitinolytic activity, and glucose (μg glucose/min/mL (**D**) or μg glucose/ mg mycelium (**E**), μg glucose /μg protein/ (**F**) for glucanolytic activity) from the appropriate substrate in 1 min. Bars represented standard deviations (SD). Values followed by different letters are significantly different (one-way analysis of variance (ANOVA) followed by a Tukey’s post hoc test with the significance evaluated at *p <* 0.05).

**Figure 7:**
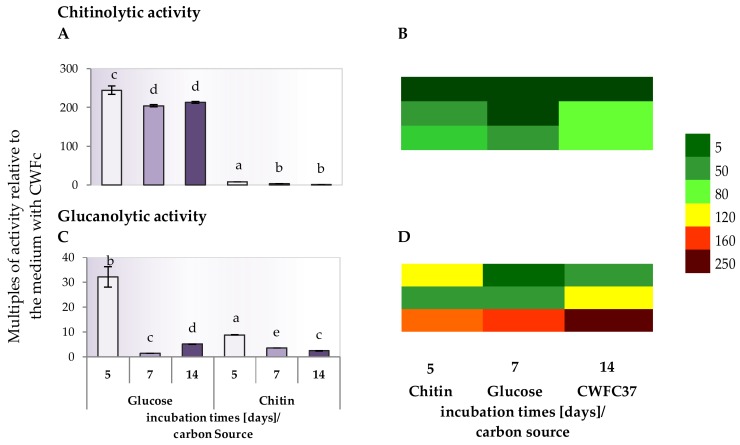
The multiples of chitinolytic (**A**) and glucanolytic (**C**) activity of *Trichoderma* DEMTkZ3A0 strain on medium supplemented with lyophilized cell wall of the pathogenic *F. culmorum* strain (CWFC37) relative to the medium with glucose or chitin. Heatmap allows to compare chitinolytic (**B**) and glucanolytic (**D**) activity on three carbon sources: Glucose, chitin and after 5, 7, and 14 days of incubation. The color scheme of the heat map fields represents the concentration in µg/mL.

The chitinolytic and glucanolytic activity of the DEMTkZ3A0 strain in the supernatants of a liquid RB mineral medium with the glucose, chitin, and lyophilized cell wall of the pathogenic *Fusarium culmorum* strain (CWFC37) as carbon sources, were determined (Figure 6A–F and Figure 7A–D).

The DEMTkZ3A0 strain demonstrated the chitinolytic and glucanolytic activity, however, glucanolytic activity was over 4-times higher than chitinolytic activity. Both activities usually increase with the time of incubation. The chitinolytic and glucanolytic activity was the highest in fluids obtained after cultivation on substrates in which the carbon source was the CWFC37. The chitinolytic activity in cultures with CWFC37 was 2–3 times higher, and the glucanolytic activity was even 10-times higher than the activity of these enzymes in cultures with other carbon sources (glucose, chitin). The DEMTkZ3A0 strain showed a higher glucanolytic activity in cultures with glucose and chitin as a carbon source compared to the chitinolytic activity (Figure 6A,B). The highest mycelium biomass and protein concentrations were found on the 5th day of incubation (Figure 6B,D). The strongest decrease in the biomass was noted on day 14 especially in glucose supplemented culture (Figure 6B,D). A gradual decrease in the protein content was observed in the culture with chitin and CWFC37 and a rapid increase in the protein concentration in the 14-day culture with glucose (Figure 6C,F). The efficiency of both enzymes converted into the biomass (Figure 6B,D) and protein (Figure 6C,F) increased with the incubation period, similar to the activity converted into mL of culture supernatant (Figure 6A,B).

The chitinolytic activity on medium with CWFC37 was about 200–250-times higher after 5, 7, and 14 day of incubation than activity of this enzyme on the medium with glucose as well as 8-times higher than on medium with chitin (Figure 7A). As for the glucanolytic activity on medium with CWFC37, it was about 30-times higher in the medium with glucose and 10-times higher in medium with chitin at 5th day of incubation (Figure 7C). Comparing the chitinolytic (Figure 7B) and glucanolytic (Figure 7D) activity in DEMTkZ3A0 strain culture, the heatmap very clearly indicates a strong activity on the CWFc37 medium and an increase in activity on the 14th day of incubation.

### 2.5. Study of the Synthesis of IAA, GA and ACC-Deaminase by Trichoderma DEMTkZ3A0 Strain Under Various Temperature Conditions and the Presence of Amino Acid Precursors

**Figure 8:**
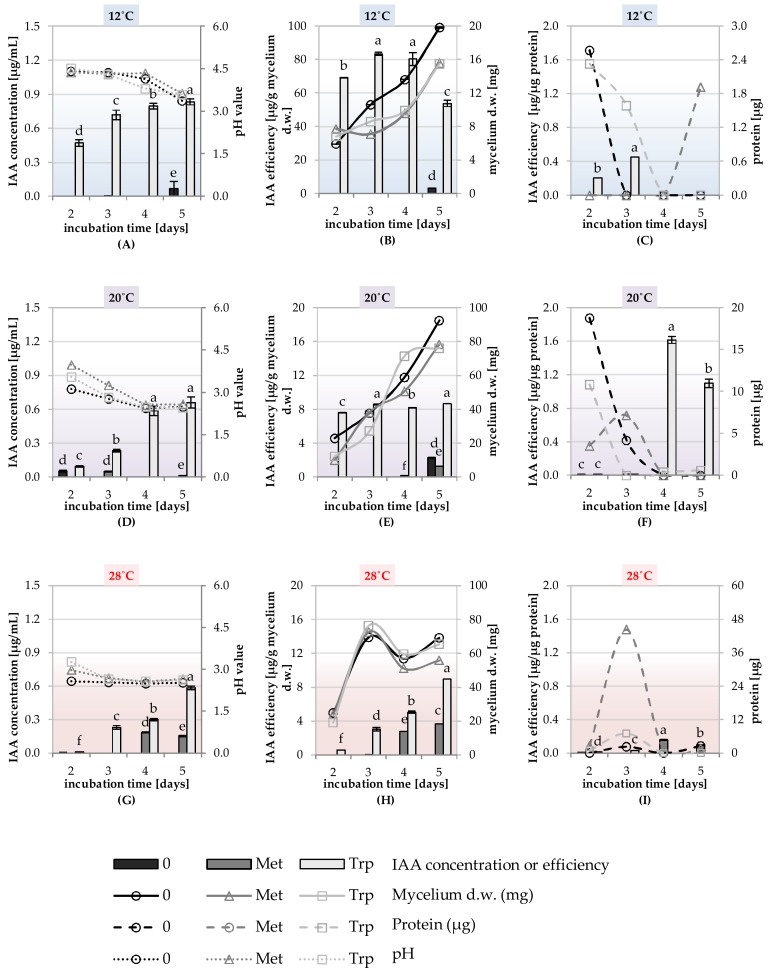
The indoleacetic acid (IAA) concentration and efficiency of IAA synthesis in *Trichoderma* DEMTkZ3A0 strain cultures grown in RB modified medium supplemented with 3.0 mM Met and Trp or without amino acids after 2, 3, 4, and 5 days incubation at 12 °C (**A**,**B**,**C**), 20 °C (**D**,**E**,**F**) and 28 °C (**G**,**H**,**I**). Bars represented standard deviations (SD). Values followed by different letters are significantly different (one-way analysis of variance (ANOVA) followed by a Tukey’s post hoc test with the significance evaluated at *p <* 0.05).

**Figure 9:**
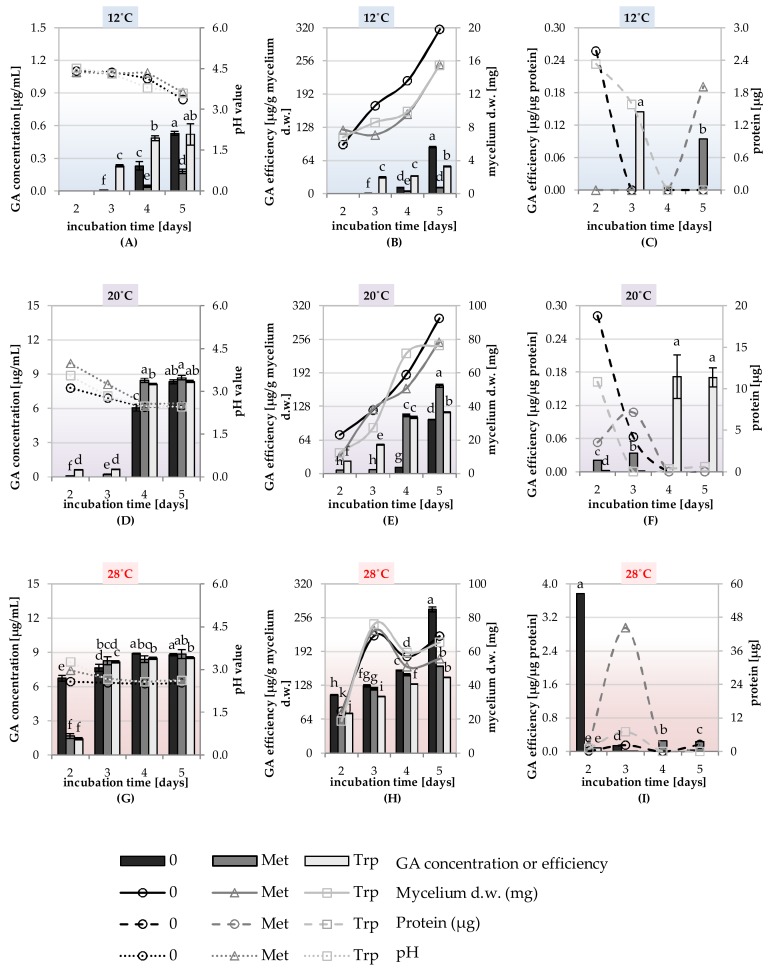
The gibberellic acid (GA) concentration and efficiency of GA synthesis in *Trichoderma* DEMTkZ3A0 strain cultures grown in RB modified medium supplemented with 3.0 mM Met and Trp or without amino acids after 2, 3, 4, and 5 days incubation at 12 °C (**A**,**B**,**C**), 20 °C (**D**,**E**,**F**) and 28 °C (**G**,**H**,**I**). Bars represented standard deviations (SD). Values followed by different letters are significantly different (one-way analysis of variance (ANOVA) followed by a Tukey’s post hoc test with the significance evaluated at *p <* 0.05).

**Figure 10:**
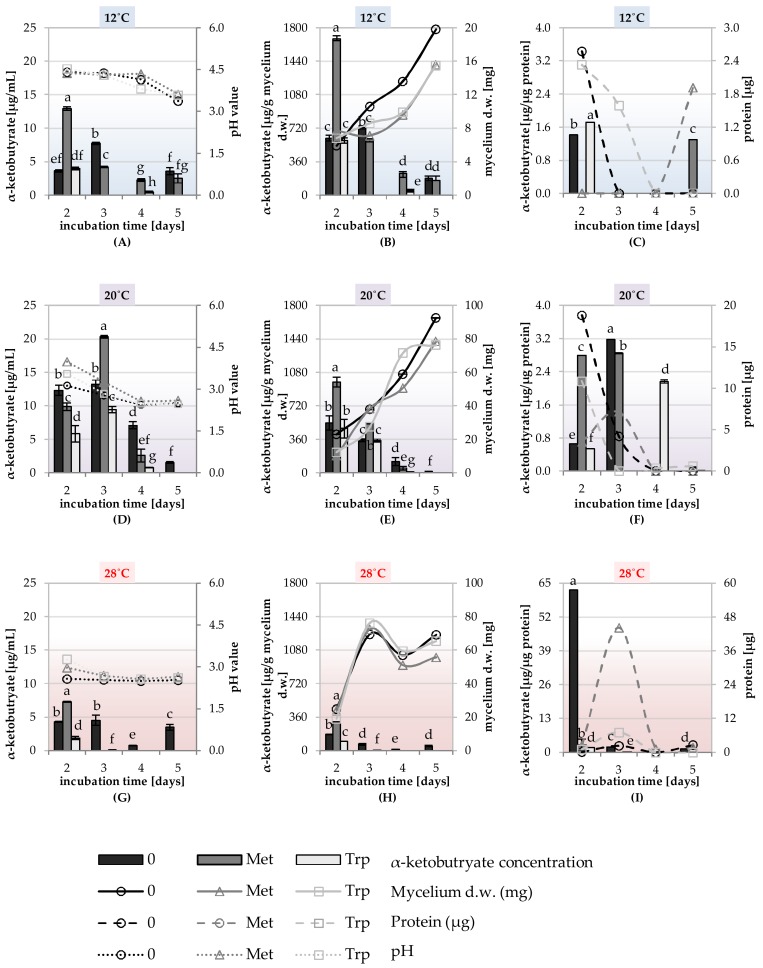
The ACC-deaminase activity and efficiency of ACC-deaminase synthesis of *Trichoderma* DEMTkZ3A0 cultures after 5 days of incubation at 12 °C (**A**,**B**,**C**), 20 °C (**D**,**E**,**F**) and 28 °C (**G**,**H**,**I**). Results are represented as nMol of α-ketobutyrate/h/mL (ACC-deaminase activity) and as nMol of α-ketobutyrate/g mycelium d.w. or nMol of α-ketobutyrate/h/mg protein (efficiency of ACC-deaminase synthesis). Bars represented standard deviations (SD). Values followed by different letters are significantly different (one-way analysis of variance (ANOVA) followed by a Tukey’s post hoc test with the significance evaluated at *p <* 0.05).

**Figure 11:**
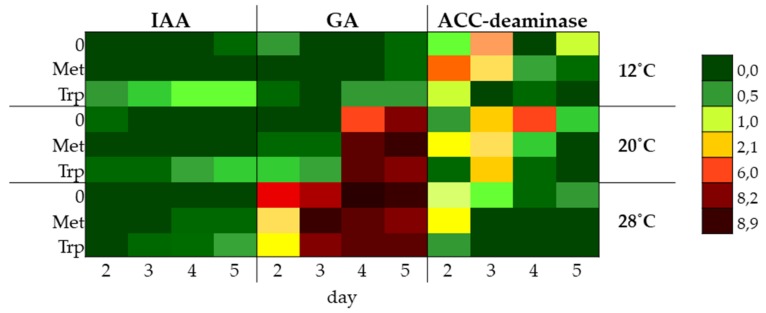
The heatmap allowed to compare IAA, GA concentration, and ACC-deaminase activity of *Trichoderma* DEMTkZ3A0 strain on RB medium with the addition of 3 mM amino acids (Met—methionine and Trp—tryptophan) at three temperatures 12, 20, 28 °C after 2, 3, 4, 5 days of incubation. The color scheme of the heatmap fields represents the concentration in µg/mL.

The ability to synthesize phytohormones IAA, GA, and enzyme–ACC deaminase was tested in the liquid shaking cultures of the DEMTkZ3A0 strain carried out on a modified mineral RB supplemented with glucose (1%) as the only carbon source and the 3mM amino acid precursors of GA and IAA phytohormones: Methionine and tryptophan. The cultures were incubated at 12, 20, and 28 °C at 60% humidity for 2, 3, 4, and 5 days (Figure 8A–H, Figure 9A–H, Figure 10A–H, and Figure 11).

The IAA auxin was detected in the culture fluids of the DEMTkZ3A0 strain obtained in four tested periods and three incubation temperatures on the medium supplemented with tryptophan as an amino acid precursor and glucose as a carbon source, where the concentration of IAA was strongly positively correlated (*R* > 0.9) with the incubation time. It was found that at 12 °C the DEMTkZ3A0 strain synthesized IAA at several times higher concentration than at 20 and 28 °C. The yield of IAA synthesis (μg IAA/g mycelium d.w.) was 10-times and 100-times higher at 12 °C than at 20 and 28 °C, respectively (Figure 8).

The concentration of GA in the culture fluids of the DEMTkZ3A0 strain was slightly dependent on the presence of the amino acid precursor in the medium. On the other hand, a very strong positive correlation between GA concentration and dry weight of mycelium as well as a negative correlation with incubation temperature was demonstrated. At a temperature of 20 and 28 °C, GA concentration reached about 9.0 μg/mL, and at 12 °C it was almost 20-times lower (about 0.5 μg/mL) (Figure 9).

The ACC deaminase activity was not dependent on the presence of the amino acid precursor, but was dependent on the time of incubation and temperature, with the highest activity of this enzyme found in 5 day cultures incubated at 12 °C. The ACC deaminase activity calculated on the dry weight of mycelium was 6-times higher in fluids obtained from cultures incubated at 12 °C than from cultures incubated at 20 and 28 °C (Figure 10 and Figure 11).

#### Principal Component Analysis (PCA) for the Physical of the *Trichoderma* DEMTkZ3A0 Strain Growing Parameters and IAA, GA Concentration, and ACC-Deaminase Activity

**Figure 12:**
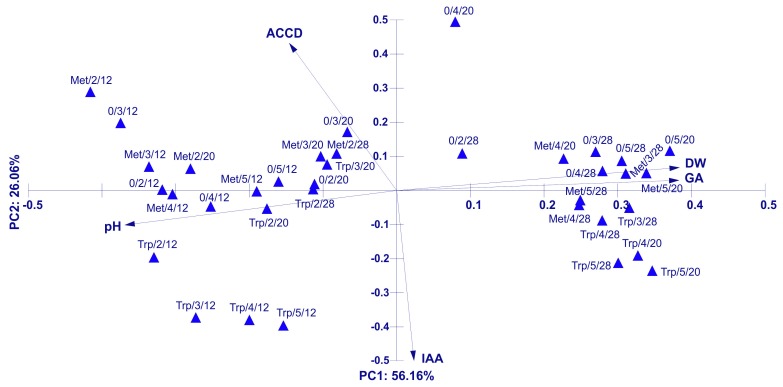
The ordination diagram showing the result of the principal component analysis (PCA) for the physical parameters of the *Trichoderma* DEMTkZ3A0 strain growing on the medium without amino acid (0) and with two amino acids: tryptophan (Trp) or methionine (Met) analyzed in four days of incubation (2, 3, 4, and 5 days) at three temperatures (12, 20, 28 °C). ACCD—average activity of ACC-deaminase; IAA—average concentration of indole acetic acid; GA—average concentration of gibberellic acid; DW—dry weight of mycelium.

The conducted analysis (Figure 12) of the main components explained 98.2% of the variability of the analyzed factors for all 4 axes, including the first axis of 56.16%, and the second one of 26.06%. The statistically significant variables related to the first axis included positively correlated DW and negatively correlated pH and ACCD. The first axis of the diagram was determined by the DW gradient. On the right side of the diagram the samples of the largest values of this factor were grouped, and on the left, with the lowest ones. The opposite orientation showed the pH vector. The samples lying on the left side of the diagram were characterized by the highest pH values, which decreased for the samples located on the right side of the diagram. The factor that also differentiated the analyzed samples was the temperature. The samples located on the left side of the diagram represented lower temperatures compared to those on the right. The statistically significant variables for the second axis of the PCA were ACCD and DW, positively correlated with this axis, and IAA and pH, negatively.

### 2.6. Promotion of Winter Wheat Seeds Growth and Activity of Plant Resistance Induction Markers

**Figure 13:**
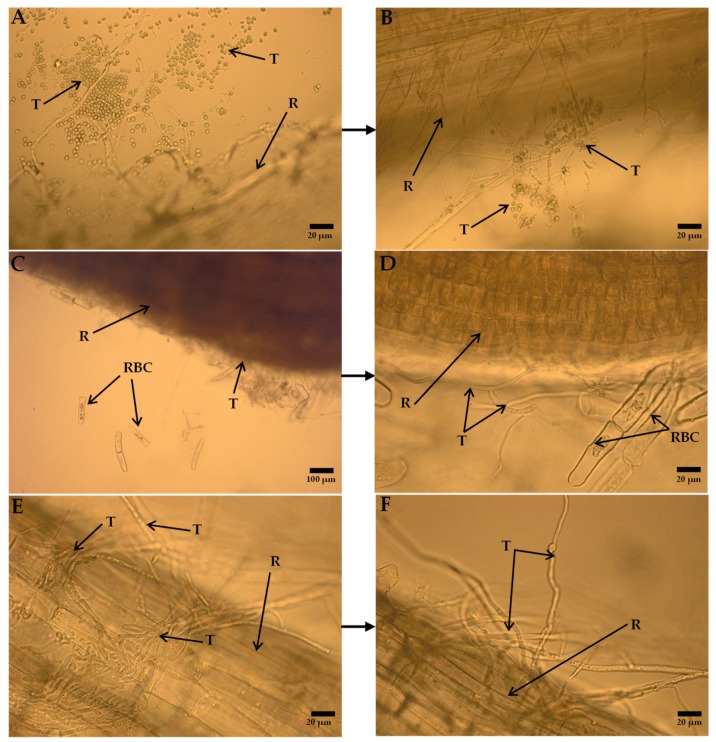
The LM micrograph of wheat seedling root colonization in hair root and RBC zone: *Trichoderma* DEMTkZ3A0 strain conidia germinated on root surface 5-day after inoculation (**A**,**B**); hyphae of *Trichoderma* DEMTkZ3A0 strain along root surface, root hairs and around root border cells (RBC) 10-day after inoculation (**C**,**D**); intensive mycelial growth of *Trichoderma* DEMTkZ3A0 strain on root surface 10 days after inoculation (**E**,**F**). Abbreviations: T—*Trichoderma* DEMTkZ3A0 strain hyphae or conidia; R—wheat root; RBC—root border cells.

**Figure 14:**
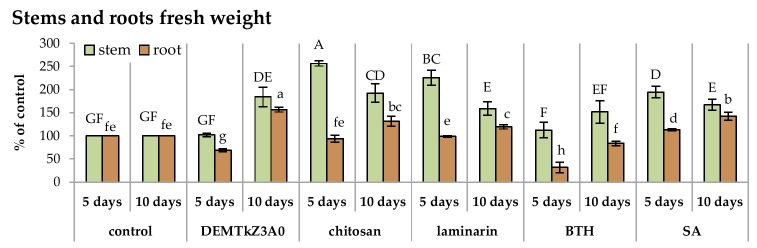
The fresh weight of wheat seedlings stem and root expressed as % of non-inoculated control after 5 and 10 days inoculation with *Trichoderma* DEMTkZ3A0 strain and potential inducers (commercial elicitors) of plant resistance: Chitosan, laminarin, BTH, and SA.

**Figure 15:**
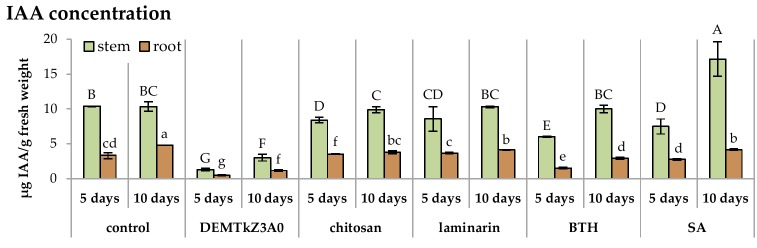
The indoleacetic acid concentration (IAA) in stem and root of wheat seedlings after 5 and 10 days inoculation with *Trichoderma* DEMTkZ3A0 strain and potential inducers (commercial elicitors) of plant resistance: Chitosan, laminarin, BTH, and SA.

The DEMTkZ3A0 strain conidia germinated on root surface of wheat seedlings and very intensive lengthening growth of this strain hyphae in the following days of incubation was observed (Figure 13A–F). The hyphae were laid along the roots and root hairs entwined and colonized root border cells (Figure 13C,D). After a 10-day incubation, they formed a dense, compact mycelium on the root surface (Figure 13E,F). After this incubation period, very significant growth of the biomass stem (it constituted 200% of the non-inoculated control) and root (150% control) was observed. Similar values were noted for chitosan and SA (Figure 14). The reduction of IAA concentration at several times was observed in inoculated and colonized roots and stems relative to non-inoculated control plants as well as plants treated with potential resistance inducers (Figure 15).

**Figure 16:**
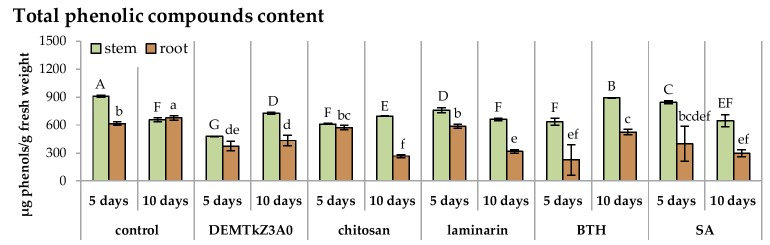
Total phenolic compounds content stem and root of wheat seedlings after 5 and 10 days inoculation with *Trichoderma* DEMTkZ3A0 strain and potential inducers (commercial elicitors) of plant resistance: chitosan, laminarin, BTH and SA.

The concentration of phenolic compounds in the plants colonized by the DEMTkZ3A0 strain or treated with commercial elicitors varied slightly as compared to the water control. An increase in the concentration was observed only in the stems of 10-day seedlings inoculated with the DEMTkZ3A0 strain conidia as well as commercial elicitors: Chitosan and BTH (Figure 16).

**Figure 17:**
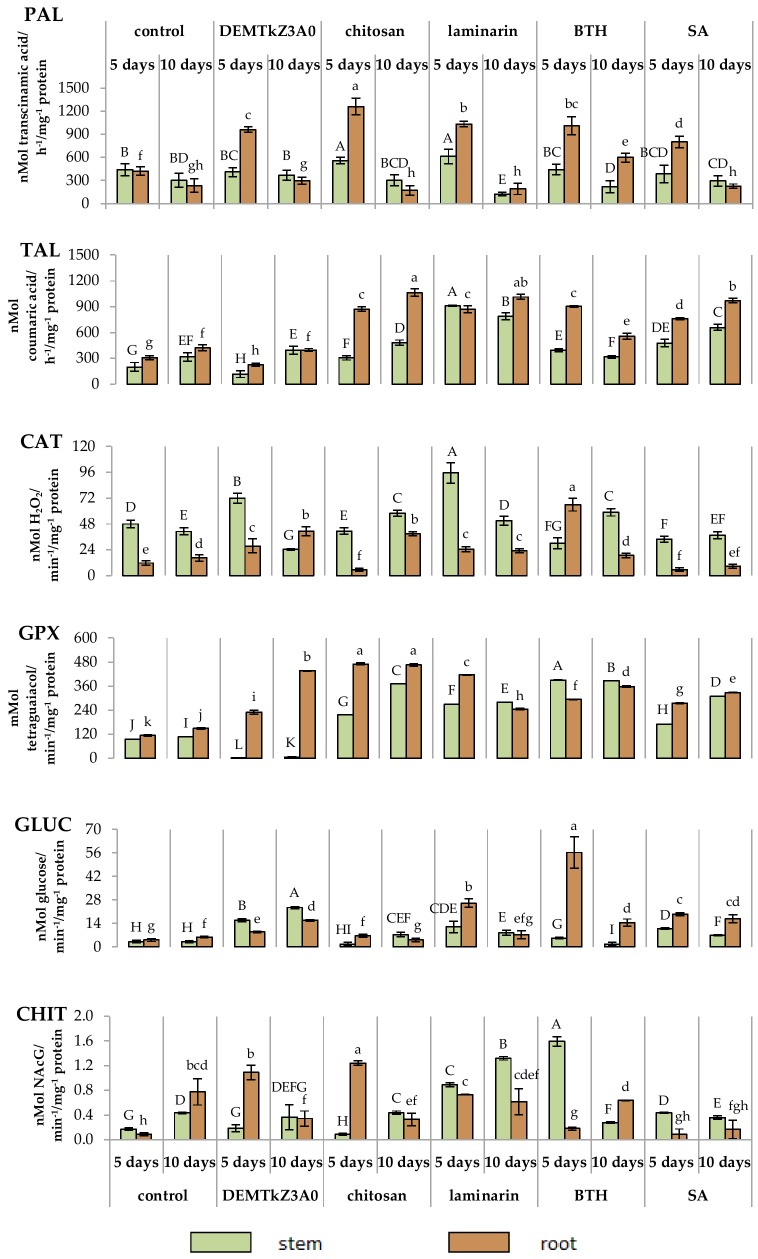
The activity of plant resistance markers (PAL—phenylalanine lyase, TAL—tyrosine lyase, CAT—catalase, GPX—guaiacol peroxidase, GLUC—glucanase, CHIT—chitinase) in the stems and roots of wheat after 5 and 10 days inoculation with *Trichoderma* DEMTkZ3A0 strain and potential inducers od plant resistance: Chitosan, laminarin, BTH, and SA.

The activity of the plant resistance induction markers (Figure 17), include enzymes of the phenylpropanoid pathway: Phenylalanine (PAL) and tyrosine lyase (TAL), catalase (CAT), and PR proteins with guaiacol peroxidase (GPX), chitinase (CHIT) and glucanase (GLUC) activities in the roots and stems of winter wheat seedlings was studied. The resistance was induced with the suspensions of conidia of DEMTkZ3A0 strain as well as the commercial elicitors with chitosan, laminarin, BTH, and SA (positive controls) introduced onto surface-sterilized wheat seeds incubated in the dark for 5 days at 20 °C.

**Figure 18:**
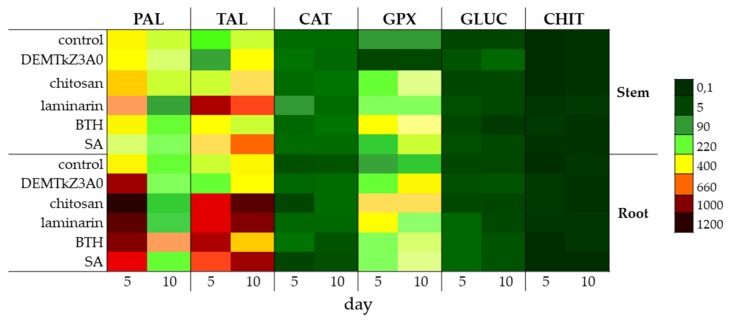
The heatmap allowed to compare PAL—phenylalanine lyase, TAL—tyrosine lyase, CAT—catalase, GPX—guaiacol peroxidase, GLUC—glucanase, CHIT—chitinase activity in the stems and roots of wheat inoculated with *Trichoderma* DEMTkZ3A0 strain or treated with commercial elicitors after 5 and 10 days of incubation. The color scheme of the heat map fields represents the concentration in nMol/mg protein.

In the wheat tissues induced with conidia, an increase in the activity of resistance markers was observed compared to both the water control and positive controls. GLUC and CHIT activity in the induced tissues was about 10-times higher than in the water control. The activity of GLUC, PAL, and GPX was higher in roots than in stems, while CHIT, TAL, and CAT were higher in stems than in roots. The activity of individual markers in the tissues of the roots and stems of wheat seedlings induced by the DEMTkZ3A0 strain was even 2-times higher than in the respective tissues of the positive controls induced by chitosan or laminarin (Figure 18).

#### Principal Component Analysis (PCA) for Activity of Plant Resistance Markers, IAA, and TPC Concentration in Wheat

**Figure 19:**
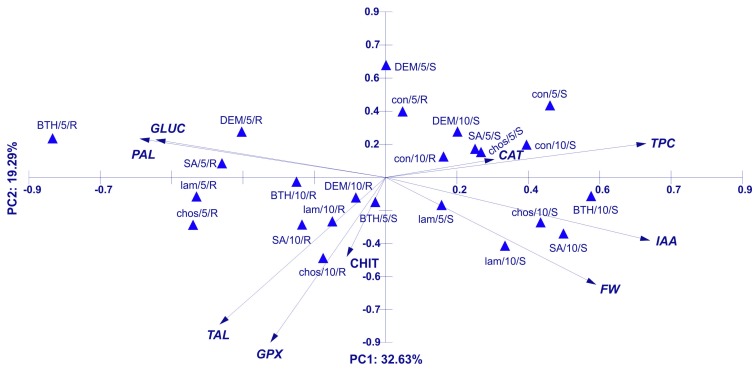
The ordination diagram showing the result of the PCA for the in vivo study parameters of the stems (S) and roots (R) of wheat seeds inoculated with the *Trichoderma* DEMTkZ3A0 strain conidia (DEM) and commercial elicitors: Chitosan (chos) and laminarin (lam) and non-inoculated (con) analyzed in 5 days of incubation at 20 °C. IAA–average concentration of indoleacetic acid; TPC—total phenolic compounds, CHIT—average activity of chitinases; GLUC—average activity of glucanases; PAL—average activity of phenylalanine ammonia lyase; TAL—average activity of tyrosine phenol-lyase; CAT—average activity of catalase; GPX—average activity of guaiacolic peroxidase; FW—fresh weight of stems and roots.

PCA (Figure 19) for in vivo assays explained 79.7% of the variability of the analyzed factors. The first axis was 32.63%, while the second one was 19.29%. Most of the variables analyzed were statistically significant for the first two axes of the diagram, except for CHIT for the first axis and CAT for the second axis. The most strongly positively correlated with the first axis were IAA, TPC, and FW, which also exhibited mutual correlation. The most negatively correlated with the discussed axis were PAL, GLUC, and TAL. PAL and GLUC were strongly correlated with each other, similarly to TAL with GPX, which also had a negative correlation with the first axis. The second axis of the diagram was positively correlated with PAL, GLUC, and TPC, and strongly negatively with GPX, TAL, FW. The analyzed samples were divided into two distinct groups. On the left side of the PCA diagram, the root samples were concentrated. Factors that affected them were PAL, GLUC, TAL, and GPX. On the right side of the diagram were stem samples. Factors associated with them were TPC, IAA, and FW.

## 3. Discussion

The root-associated microorganisms are mainly recruited from the surrounding soil in the rhizosphere (a thin layer of soil around roots) [34,38,91,92] and the rhizoplane (root surface). In internal root tissues (endosphere), they form microbial communities that are very different from microbial communities colonizing the adjacent soil [93,94]. As for fungi of the *Trichoderma* genus, the ability to colonize surface root tissues was demonstrated, but mainly in the root hair and elongation zone [21]. The lack of information on the colonization by these fungi of the apical zone and located in root border cells (RBCs) zone is noticed [91,95]. RBCs play an important role in the interaction between roots and rhizosphere/rhizoplane/soil colonizing microorganisms and in plant defense against phytopathogens and many abiotic factors [96,97], operating not only physically by trapping phytopathogens, but also through numerous specialized defense mechanisms: They can repel and bind bacteria [98], attract and immobilize parasitic nematodes [99], and create a mantle-like structure acting as a decoy for pathogenic fungi [91,100]. The DEMTkZ3A0 strain very effectively colonized not only surface tissues in the root hair and elongation zone, but also wheat RBCs, which indicates the possibility of an additional mechanism of indirect influence of DEMTkZ3A0 on phytopathogens via RBC.

The beneficial microbes can produce plant hormones and use them to promote plant immunity and determine the community composition [101], thus adding another level of complexity to the hormone crosstalk in plant–microbe interactions. In the case of phytopathogen infection, plants recruit microorganisms from their ectosphere [102], which initiates changes in host plant immunity primed for intensified induction [48,102,103,104]. A focus on the molecular dialogue between the plant holobiont–plant and their microbiome (endosphere and ectosphere microbial populations) is important for better understanding how microorganisms can live on or in their host plants [63]. The certain root endophytes were shown to be able to limit or change plant hormone signaling in different ways, e.g., by synthesizing auxins and auxin analogs along with gibberellins (GAs) [105,106], which probably attenuate SA signaling [70].

Several reports have been made on PGP fungi having one of the important properties conditioning the ability to promote plant growth, direct or indirect inhibition of phytopathogen growth, and little about the strain combining many of the features important for this process. The *Trichoderma* spp. possesses a wide variety of biotrophic and saprotrophic ecological strategies ranging from parasitism or predation to saprotrophy, and their ability to parasitize other fungi (mycoparasitism) offers great potential in a biological control against soil-borne fungal diseases [107].

A number of effective strains have been found among the genus *Trichoderma* spp., but no data is available on strains of the *T. koningiopsis* species isolated from the rhizosphere of healthy cereal plants. The research subject has become the DEMTkZ3A0 strain, initially classified to the species *T. koningiopsis* based on ITS analysis, that combines a wide variety of activities ranging from strong mycoparasitic properties to phytopathogens of the genus *Fusarium* spp., the ability to synthesize CWDE, phytohormones and ACC deaminases, as well as inducing plant resistance. CWDE (chitinase and glucanase) is produced by DEMTkZ3A0, both by direct degradation of phytopathogen CW and indirectly by releasing from the CW of the pathogen as well as its own CW (during autolysis) fragments with elicitor properties. Altogether, our results suggest that there are multiple mechanisms and molecules involved in a plant growth promotion by the *Trichoderma* DEMTkZ3A0 strain affected by the environmental conditions.

Although many ways of regulating the physiological processes in plants are known, very little attention has yet been given to the interaction between defense hormones and growth hormones [108]. The crosstalk between the phytohormonal and signaling defensive pathways may occur [76,77].

The level of activity of plant resistance markers in the DEMTkZ3A0 inoculated plants varied over time and developed differently in the stem and root. The inoculation of wheat grain with the DEMTkZ3A0 strain led to an increase in the activity of the main marker enzyme opening the phenylpropanoid pathway–phenylalanine lyase (PAL) in the roots of 5-day-old seedlings. In 5-day-old seedlings, the level of oxidative enzymes—catalase in the stems and peroxidase in the roots—increased more than 2-times. In the roots of these seedlings, the concentration of protein with the properties of chitinase belonging to pathogenesis proteins PR was also very strongly increased. The level of other PR protein with glucanase activity increased several times in a relation to the negative control (not inoculated or not treated with commercial elicitors) in the stems as well as in the roots of 5- and 10-day-old seedlings. In the roots of 10-day-old seedlings, the activity of protein with peroxidase properties increased up to 3-times. It should be emphasized that the effect of resistance markers induction by the tested DEMTkZ3A0 strain was comparable and often much higher than that obtained in positive controls treated with commercial elicitors: Chitosan, laminarin, BTH, or SA. It is worth noting that the inoculation with the tested DEMTkZ3A0, similarly to treatment with commercial elicitors, had the same effect on the level of phenolic compounds in the roots and stems of seedlings. The concentration of phenols in the roots usually decreased slightly but statistically significant increase was noted in the stems of 10-day DEMTkZ3A0 inoculated seedlings similar to those treated with chitosan and BTH.

*Trichoderma harzianum* isolated from soybean promoted growth of soybean seedlings, induced resistance against *F. oxysporum* by the increase of reactive oxygen species (ROS) scavenging enzymes, such as peroxidase and superoxide dismutase (SOD) [80]. After inoculation of wheat seedlings with *T. longibrachium* T6 strain, in salt stress condition, the antioxidant enzymes (SOD, peroxidase and catalase) activity increased about, 30%, 40%, and 20%, respectively [109]. However, the application of a bacterial isolate from Spitsbergen on *Phaseolus coccineus* before Cu administration resulted in the only increase of SOD activity [92].

A very clear difference in the level of IAA concentration determined in the seedling stems and roots was observed between seedlings treated with commercial elicitors and seedlings inoculated with the DEMTkZ3A0. In the inoculated plants by this strain, there was a significant decrease in the concentration of IAA several times, while in the seedlings treated with elicitors it remained at the control level (only in 10-day SA-treated stems increased twice) and in the roots treated with elicitors it decreased slightly.

This may suggest a significant interaction between phytohormonal and plant resistance pathways, in particular after inoculation with a strain, that has the ability to induce immunity and at the same time to synthesize its own phytohormones and an enzyme (ACC deaminase) that regulates ethylene levels. It is most likely that the cross-talk between immune hormones (such as SA, JA, and ethylene) and growth hormones occurs in this interaction. A positive effect of JA positively and a negative impact of SA on the level of auxin in resistance to necrotrophic and biotrophic pathogens were observed, and a significant increase in the endogenous IAA level has been reported upon pathogen infection [108].

On the one hand, increased levels of endogenous plant IAA have been observed during pathogen infection, but on the other side, a few detailed studies reviewed by Fu and Wang [110] have revealed that auxin can enhance disease resistance. A number of studies attempt to explain the influence of both PGP fungal and bacterial strains and phytopathogens (e.g., gall and tumor-inducing) on the IAA production [3,5,110]. Most reports indicate that an excessive increase in the IAA concentration in the plant tissue is one of the major pathogenicity factors of bacterial and fungal phytopathogens. Auxin signalling is intimately connecting with the plant growth and development as well as with plant resistance and can be modified by plant colonizing microorganisms, because plants might receive microbial IAA by own auxin receptors [110].

The research conducted on a cucumber indicated that higher synthesis of the IAA by the putative mutant of *Trichoderma harzianum* may explain the enhancement of rhizosphere soil, rhizoplane as well as roots, and stems colonization [14,28]. The *Trichoderma asperellum* Q1 strain with the ability to synthesize IAA, GA, and abscisic acid (ABA) in a liquid medium also affect an increase of these phytohormones concentration in cucumber seedlings leaves and root growth [111].

The production of IAA by fungi belong to *Trichoderma* genus is dependent on strain and diverse external stimuli are associated with its production [112]. The concentration of IAA and GA in the DEMTkZ3A0 cultures and the efficiency of their synthesis in terms of mycelium biomass or protein had a clear upward trend during the 5-day culture. Such a tendency was also observed in the cultures of Zygomycota fungal strains belonging to two *Mortierella* species: *M. antarctica* and *M. verticillata*; yet, it is surprising that IAA synthesis in these psychrophic strains was positively correlated with temperature and was the highest at 20 °C [81] and bacterial isolate (S17) from Spitsbergen [92]. The non-pathogenic PGPF *Fusarium culmorum* strain synthesized higher amounts of IAA and GA than DRMO and pathogenic strains. Amino acid precursors (methionine and tryptophan) exerted a positive effect on the synthesis of these phytohormones [3]. The IAA synthesis in the DEMTkZ3A0 cultures was determined by the presence of the amino acid precursor tryptophan, and IAA was found in cultures without this precursor in only a few cases. Such dependence of IAA synthesis on tryptophan and its concentration was also found in *Mortierella* spp. cultures [81]. Tryptophan had a positive effect on GA synthesis by the DEMTkZ3A0 only at the low temperature (12 °C); at the higher temperatures, the addition of amino acids slightly influenced the synthesis of this hormone.

In contrast, methionine in some DEMTkZ3A0 cultures stimulated ACC deaminase activity. However, in the case of ACC deaminase activity, a clear decrease in the activity of this regulatory enzyme and in the efficiency of its production was observed with the age of culture, whereas the highest activity and efficiency of the production of this enzyme was noted in young cultures. The deaminase activity was the highest in the DEMTkZ3A0 cultures incubated at 20 °C, whereas for *Mortierella* spp., deaminase activity was the highest in cultures incubated at 9 °C [81].

A similar increase of the chitinolytic and glucanolytic activity observed in the DEMTkZ3A0 cultures along with the incubation time, despite the significant decrease in the biomass in the long-term cultures. This demonstrates the autolytic nature of the produced enzymes, which is consistent with the results obtained for CWDE complexes produced by pathogenic and non-pathogenic strains of the *Fusarium* spp., whose strong degradation capabilities of both fungal and plant cell walls were demonstrated [56,57]. In the structure of the fungal CW (FCW), two separated layers are clearly visible: (1) The inner FCW built of a chitin–glucan-rich interconnected matrix and (2) the outer FCW rich in mannosylated glycoproteins [113]. FCWs play the main role in the plant defense elicitation process by the release of oligomers of chitin and β-glucan from the FCW acting as PAMPs during infection [114,115,116], resulting in activation of PAMP-triggered immunity (PTI) [117,118,119,120,121]. In several recent studies, not only CW oligomers but various proteins and peptides from *Trichoderma* were shown to induce the host defense responses [122]. The proteins with chitinolytic and glucanolytic activity synthesized by the DEMTkZ3A0 may also act as an elicitor independent on the enzymatic activity, as was demonstrated by Sharon et al. [123] for the xylanase synthesis by the *T. viride* strain.

In the triple interaction of *Trichoderma–Fusarium–*plant, the synthesis of chitinase may be limited by the toxins of *Fusarium* spp. The DON production by *F. culmorum* and *F. graminearum* strains was reported to repress expression of the chitinase gene (encoding the *N*-acetyl-β-d-glucosaminidase) in *Trichoderma atroviride* [124].

*T. atroviride* [125] in co-culture with *B. ciner*ea CW resulted in the secretion of the CW hydrolytic enzymes and antibiotic fractions of peptaibols, which inhibited *B. cinerea* spore germination, causing a fungicidal effect [122]. Peptaibols were shown to induce plant resistance and auxin production and disruption of the auxin response gradient in root tips [82].

Taking into account the parameters determined in vivo, the weight of stems and roots of wheat seedlings and the activity of resistance marker enzymes, PCA analysis showed a very strong relationship between PAL and glucanase activity in the roots of 5-day DEMTkZ3A0 inoculated seedlings. The factors connected with stems and roots were differently correlated and inversely proportional, which means that both parts of the plant variously react to the same agent. The activity of PAL was dependent on peroxidase activity and similar relationship was connected with both PR proteins glucanase and chitinase.

PCA analysis determined in vitro suggests that the synthesis by the tested DEMTkZ3A0 strain of both tested phytohormones (IAA and GA) and the synthesis of ACC deaminase are independent on each other. The IAA synthesis in DEMTkZ3A0 strain cultures was strongly stimulated with tryptophan and low (12 °C) temperature, whereas GA synthesis was dependent on the fungal biomass and, like ACC deaminase synthesis, was more effective at higher (20, 28 °C) temperatures. On the basis of PCA results, we can affirm differences among culture supplemented with methionine and tryptophan. DEMTkZ3A0 biomass and GA were correlated with each other and dependent on temperature level, thus the effectiveness of production both of them was temperature dependent.

Most *Trichoderma* strains are mesophilic with the greatest saprophytic activity from 15 to 21 °C [40,85]. They cannot protect germinating seeds from soil-borne diseases caused by cold-tolerant strains of plant pathogenic fungi during cold autumn and spring conditions. Although all the species of *Trichoderma* studied by Singh et al. [126] produced sufficient biomass at different temperatures in the range of 20–35 °C but they were found to be the best grown at a temperature range of 25 to 30 °C. The optimum temperature for *Trichoderma* mycelial growth was observed between 24 and 28 °C, however, in the range of 15 to 24 °C, the temperature was positively correlated with growth rates, followed by a plateau, and the maximum temperature for *Trichoderma* spp. growth was examined at 32 °C [127]. The results obtained for the DEMTkZ3A0 cultures indicate very high adaptability to temperature conditions of the tested strain, especially in the presence of glucose in the medium. At 12 °C, this strain showed particularly strong ability to synthesize auxin and high ACC deaminase activity, while the GA concentration in the DEMTkZ3A0 cultures was positively correlated with the temperature value.

The tested *Trichoderma* DEMTkZ3A0 strain strongly (almost by 100%) limited the growth of *Fusarium* spp. phytopathogens in in vitro tests and have strong chitinolytic and glucanolytic activity in culture with *F. culmorum* CW. *Trichoderma harzianum* mycoparasitic strains shown similarly high frequency in coiling around Basidiomycota phytopathogenic fungus *Rhizoctonia solani* hyphae but correlation between coiling frequency and chitinase and glucanase activity was not found [128]. The mycoparasitic activity of DEMTkZ3A0 strain was not limited to hyphae of *Fusarium* strains. It was observed that hyphae of the *Trichoderma* DEMTkZ3A0 strain coiling around the spores (phialides with macroconidia) of the pathogenic DEMFc37 strain, not yet reported in the literature.

The obtained results indicate the mycoparasitic DEMTkZ3A0 strain to have a positive effect on the plant growth and its protection against fusariosis through phytohormonal interaction and regulating the level of growth and immune hormones (e.g., IAA and ethylene), a direct mechanism of mycoparasitism enhanced by a strong chitinolytic and glucanolytic activity and probably synthesis of other secondary metabolites with antibiotic properties and primarily by inducing pathways of plant resistance.

## 4. Materials and Methods

### 4.1. PCR Amplification and Sequencing of the Fungal ITS Region

DNA extraction procedure was performed according to the method described by Borges et al. [129]. The degree of purification and concentration of the DNA sample were estimated using ND1000 spectrophotometer (Thermo Scientific, West Palm Beach, FL, USA). Polymerase chain reaction amplifications (PCR) with primers (ITS 1 and ITS 4) were carried out according to the protocol of White et al. [130]. Reactions were done in a TPersonal thermocycler (Biometra, Germany), and purified by microfiltration using Clean-up kit (A&A Biotechnology, Poland). BigDye™ Terminator Cycle Sequencing Kit and ABI PRISM 3730 XL sequencer were used in automatic sequencing (Applied Biosystems, Carlsbad, CA, USA). The database searches were performed with the BLAST program at the National Centre for Biotechnology Information (Bethesda, MD, USA) [131]. The multiple DNA sequence alignments were performed with the Clustal-W algorithm [132]. The neighbor-joining (NJ) algorithm was employed to construct a phylogenetic tree as implemented in MEGA v.7.0 software [133]. The topology of the tree was evaluated by bootstrap analysis of the sequence data based on 500 random resamplings.

### 4.2. Fungal Isolates and Their Molecular Identification

The experiments were carried out on the *Trichoderma* DEMTkZ3A0 strain and three phytopathogenic isolates: *Fusarium graminearum* DEMFc36, *Fusarium culmorum* DEMFc37, and *Fusarium oxysporum* DEMFc38.

The DEMTkZ3A0 strain was isolated in Poland near Lublin region from winter rye (*Secale cereale* L. cv Dańkowskie Złote) rhizosphere soil. This strain was deposited at the Department of Environmental Microbiology (DEM) Fungal Collection at Maria Curie-Sklodowska University, Lublin, Poland. The DEMTkZ3A0 strain was morphologically and genetically identified according to the ITS (internal transcribed spacer) region sequencing, which was deposited in the National Centre for Biotechnology Information GeneBank (https://www.ncbi.nlm.nih.gov/) at 25 July 2018 with MH651381 sequence number.

The DEMFc36, DEMFc37 and DEMFc38 strains were isolated in Poland near Lublin region from winter wheat (*Triticum aestivum* L.) plants with severe fusariosis symptoms. These strains were deposited at the Department of Environmental Microbiology (DEM) Fungal Collection at Maria Curie-Sklodowska University, Lublin, Poland and in the Centraalbureau voor Schimmelcultures Collection (CBS), P.O. Box 85167, NL-3508 AD Utrecht, The Netherlands (https://www.cbs.knaw.nl/database/). The *Fusarium* spp. strains were morphologically and genetically identified according ITS region sequencing, which were deposited in the National Centre for Biotechnology Information GeneBank at 10 April 2006 with a DQ450878 sequence number for the DEMFc37 strain and a DQ450879 sequence number for the DEMFc38 strain. The DEMFc36 strain was deposited at 15 April 2006 with a DQ453701 sequence number.

### 4.3. Storage Conditions of Trichoderma DEMTkZ3A0 and Fusarium *spp.* Strains

The fungal *Trichoderma* and *Fusarium* strains used in the study were stored on Martin medium (glucose 10.0g; peptone 5.0g; KH_2_PO_4_ 1.0g; MgSO_4_ *×* 7H_2_O 1.0g; agar 15.0g; 1% rose bengal solution 3 mL; 1% streptomycin solution 3 mL in 1000 mL DW) slants at 4 °C in the Fungal Collection of the Department of Environmental Microbiology (DEM) at Maria Curie-Sklodowska University in Lublin, Poland.

### 4.4. Growth Rate of Trichoderma DEMTkZ3A0 Strain

To determine the optimal temperature and carbon source for the growth of the *Trichoderma* DEMTkZ3A0 strain, mycelia discs (0.8 cm) from initial cultures (grown on RB medium for 7 days at 12, 20, and 28 °C) were transferred to two modified Ryes and Byrde [90] medium (RB) with the addition of 1% glucose or 1% sucrose in petri dishes (total diameter of 9.0 cm). The petri dishes were incubated at 12, 20, and 28 °C for 12 days. After 1, 3, 5, 7, 9, and 12 days of incubation at three temperatures, the diameters of the colonies were measured and the R factor of the growth rate was calculated from the formula and was presented as mm^2^ mycelium/day^−1^.

R=[(D2)2− d2)2 · πT

where *R*—growth rate factor; *D*—diameter of the mycelium (mm); *d*—mycelia discs (8.0 mm); *π*—3,14; *T*—incubation time (day).

### 4.5. Biotic Effect and Mycoparasitic Interaction Between Trichoderma DEMTkZ3A0 Strain and Phytopathogens

The ability of the DEMTkZ3A0 strain to limit the growth of phytopathogens was determined based on the biotic effect between the *Trichoderma* DEMTkZ3A0 strain and *Fusarium* spp. DEMFc36, DEMFc37, and DEMFc38 pathogens. The biotic effect was studied on PDA medium (potato dextrose agar, Sigma-Aldrich, St. Louis, MI, USA). An agar discs (9.0 mm in diameter) overgrown with the DEMTkZ3A0 and phytopathogens mycelium were inoculated from initial cultures (grown on PDA medium for 7 days at 20 °C) on the same petri dishes with an equal distance from the centre of the plate. At the same time, control versions were prepared for each strain used. After 14 days of incubation at 20 °C, each version was evaluated according to the scale developed by Mańka [134], taking into account three parameters: (a) The degree of colony surrounding one fungal by the other, (b) inhibition zone and (c) reduction of colony compared to the control. In the case of the dominance of DEMTkZ3A0 strain, the “+” sign (positive effect) was awarded, while the pathogen was given a negative effect (sign “−“). The obtained assessment gave together an individual, total biotic effect, illustrating the influence of DEMTkZ3A0 strain on the growth on the pathogenic fungus.

On the same petri dishes, the ability of the DEMTkZ3A0 to enter into a parasitic interaction with pathogenic DEMFc36, DEMFc37, and DEMFc38 strains was determined. A characteristic image of the parasitic interaction, including chemotaxis, adhesion, formation of coils, and appressorium like-structures was observed using an Olympus BX53 Upright Microscope equipped with a Olympus XC30 camera and photographed.

### 4.6. Preparation of Trichoderma DEMTkZ3A0 Strain Conidia

The conidia used for the preparation of inocula were obtained from a culture grown on a modified RB liquid medium with 1% glucose as a carbon source. The DEMTkZ3A0 strain was cultivated in darkness at 20 C and 60% relative humidity in an Innova 4900 growth chamber (New Brunswick Scientific, Edison, NJ, USA) at 120 rpm for 7 days. Then, the cultures were filtrated through 5 layers of sterile gauze (cotton). The supernatants were collected, and the conidia were pelleted by centrifugation (Beckman J2-HS) at 10,000*× g* for 15 min. They were washed three-times by suspension in sterile distilled water and vortexed for 30 s. Then the numbers of conidia in the suspension were determined in a hemocytometer, using an Olympus BX53 Upright Microscope, and adjusted by dilution to the desired concentration (density) [3].

### 4.7. Study of the Chitinolytic and Glucanolytic Activity in Trichoderma DEMTkZ3A0 Strain Liquid Culture

The chitinolytic and glucanolytic activity was tested in a long-term liquid shaking (150 rpm) DEMTkZ3A0 cultures carried out on the RB medium composed of KH_2_PO_4_ 1.0 g, MgSO_4_ *×* 7H_2_O 0.5 g, KCl 0.5 g, (NH_4_)_2_SO_4_ 0.5 g; and 1 mL microelements solution prepared separately (composed of Na_2_B_4_O_7_ *×* 10H_2_0 100.0 mg. CuSO_4_ *×* 5H_2_0 10.0 mg, FeSO_4_ *×* 7H_2_0 50.0 mg, MnSO_4_ *×* 5H_2_O 10.0 mg, (NH_4_)_6_Mo_7_O_24_ *×* 4H_2_O 10.0 mg, ZnSO_4_ *×* 7H_2_O 70.0 mg in 100 mL DW) and streptomycin 30.0 mg in 1000 mL DW, pH 5.35; in three experimental versions that differed in the carbon source: Glucose, chitin and lyophilized cell wall of the pathogenic *Fusarium culmorum* strain (CWFC37), introduced at a concentration of 0.2%. 50 mL of the liquid RB medium in 250 mL Erlenmeyer flasks was inoculated with a conidia suspension (5.0 *×* 10^6^) of the DEMTkZ3A0 strain prepared as described before (Section 4.5). The cultures were cultivated in darkness at 20 °C and 60% relative humidity in an Innova 4900 growth chamber (New Brunswick Scientific, Edison, NJ, USA) at 120 rpm for 5, 7, and 14 days. Three cultures of DEMTkZ3A0 strain on each carbon source for each period were studied, and the experiments were repeated three times.

The glucanolytic activity was determined in supernatants after their 3 h incubation with 0.1% solution of laminarin from *Laminaria digitata* (Sigma-Aldrich, St. Louis, MI, USA) in acetate buffer (pH 5.6) with 0.02% sodium azide (NaN_3_) as well as the chitinolytic activity was determined after 2.5 h incubation of culture fluid supernatants with 0.5% suspension of colloidal chitin in phosphate buffer (pH 5.6) with 0.02% sodium azide (NaN_3_). The glucanolytic activity was determined by the Somogyi [135] and Nelson [136] method modified by Hope and Burns [137] based on the concentration of released product: glucose. The chitinolytic activity was determined by the Rodriguez-Kaban et al. [138] method, modified by Rössner [139], based on the concentration of release product: *N*-acetyl-d-glucosamine (GlcNAc). The absorbance was measured at λ = 520 nm (glucanase) and λ = 585 nm (chitinase) within 20 min using the Varian Cary 1E UV–visible Spectrophotometer. Chitinolytic and glucanolytic activity expressed as U per mL of supernatant was calculated from the linear portion of activity curves. One unit of chitinolytic activity was defined as the amount causing the liberation of 1 μg per min of GlcNAc equivalent from chitin as well as one unit of glucanolytic activity was defined as the amount causing the liberation of 1 μg per min of glucose equivalent from laminarin. In addition, chitinolytic and glucanolytic activity was expressed as μg of GlcNAc/mg of dry weight (d.w.) of mycelium and as μg of GlcNAc/μg of protein. The protein concentration was quantified by the Bradford method [140].

### 4.8. Study of the Synthesis of IAA, GA and ACC-deaminase in Trichoderma DEMTkZ3A0 Strain Liquid Culture

To determine the IAA, GA and ACC-deaminase concentrations, the DEMTKz3A0 isolate inoculum (1.0 *×* 10^5^ conidia per mL) prepared as described before (Section 4.5) was cultivated in 100 mL Erlenmeyer flasks with 20 mL of a modified RB medium supplemented with solutions of glucose (1%) as a carbon source, and the phytohormone precursors tryptophan and methionine (3 mM) sterilized using syringe filters (0.22 μm) at 12, 20, and 28 °C and 60% relative humidity in an Innova 4900 growth chamber (New Brunswick Scientific, Edison, NJ, USA) at 120 rpm for 2, 3, 4, and 5 days. The dry weight of mycelium (mycelium d.w.) was determined after collecting on Whatman no. 1 filters and weighting after drying at 65 °C for 24 h. To determine the ability of the DEMTzZ3A0 strain to synthesize IAA, GA, and deaminase ACC the supernatants of the liquid culture were additionally centrifuged (10,000*× g* for 10 min).

#### 4.8.1. Determination of IAA Concentration

The IAA concentration in the culture supernatant was estimated according to the method of Glickmann and Dessaux [141] using Salkowski’s [142] reagent, modified by Pilet and Chollet [143]. An aliquot of 1 mL of the culture supernatant was mixed with 1 mL of Salkowski’s reagent. The mixture was intensively mixed (Heidolph REAX top vortex). The absorbance of the pink color developed after 30 min of incubation in darkness at 20 °C was read at *λ* = 530 nm using a Varian Cary 1E UV–visible Spectrophotometer. The IAA concentration in the cultures was determined using a calibration curve of pure IAA (Sigma-Aldrich, St. Louis, MI, USA) as a standard following the linear regression analysis. The IAA concentration was expressed as μg of IAA/mL of culture supernatants and as μg of IAA/g of mycelium d.w as well as μg of IAA/μg of protein. The protein concentration was quantified by the Bradford method [140].

#### 4.8.2. Determination of GA Concentration

The GA concentration was determined using a modified method described by Bruckner et al. [144,145], Hasan [146], and Tien et al. [147]. The culture supernatants were adjusted to pH 2.8 using 1 M HCl and then extracted three-times with an equal volume of ethyl acetate. The ethyl acetate extracts were pooled and vacuum-evaporated in a rotary evaporator at 20 °C (Eppendorf Concentrator plus). The dry pellet was dissolved in the ethanol:H_2_SO_4_ (90:10) mixture. The absorbance was measured at *λ* = 254 nm in quartz cuvettes using the Varian Cary 1E UV–visible Spectrophotometer. The GA concentration in the cultures was determined using a calibration curve of pure GA (Sigma-Aldrich, St. Louis, MI, USA) as a standard following linear regression analysis, and was expressed as μg of GA/mL of culture supernatants and μg of GA/g of mycelium d.w. as well as μg of GA/μg of protein. The protein concentration was quantified by the Bradford method [140].

#### 4.8.3. Determination of ACC-Deaminase Activity

The ACC deaminase concentration was determined using method described by Kumar et al. [133]. An aliquot of 200 µl of the culture supernatant was mixed with 25 µl of toluene. The mixture was intensively mixed (Heidolph REAX top vortex). 20 µl of 0.5 M ACC (1-aminocyclopropane-1-carboxylic acid) was added to the mixture and incubated for 15 min at 30 °C. Then 200 μL of 0.56 N HCl was added to the mixtures and centrifuged at 10,000*× g* for 15 min. Then 60 µL of a solution of 2,4-dinitrobenzyl hydrazine (prepared by dissolving 0.2 g of 2,4-dinitrophenylhydrazine in 100 mL of 2 N HCl) was added. The mixture was incubated for 30 min at 30 °C and then 400 μL of 2 N NaOH was added and intensively mixed (Heidolph REAX top vortex). The absorbance was measured at *λ* = 540 nm using the Varian Cary 1E UV–visible Spectrophotometer. The ACC deaminase concentration in the cultures was determined using a calibration curve of pure α-ketobutyrate (Sigma-Aldrich, St. Louis, MI, USA) as a standard following linear regression analysis, and was expressed as μg of α-ketobutyrate/mL of culture supernatants, μg of α-ketobutyrate/g of mycelium d.w., and μg of α-ketobutyrate/μg of protein, released during the hour of incubation. The protein concentration was quantified by the Bradford method [140].

### 4.9. In Vivo Stimulation of Wheat Growth and Analysis of the Activity of Plant Resistance Markers

#### 4.9.1. Preparation of Seeds

The seeds of winter wheat (*Triticum aestivum* L. cv Arkadia) purchased from DANKO Plant breeding (Choryń, Poland) were used in experiments. The seeds were surface-sterilized according to the procedure described by Jaroszuk-Ściseł and Kurek [57]. For 30 min, the grains were washed in tap water, taken soaked for 10 min in a solution of HgCl_2_ (0.1%, *w*/*v*), submerged for 10 min in a solution of H_2_O_2_ (30%, *w*/*v*), and washed three times with sterile deionized water. Surface-sterilized seeds were transferred to sterile petri dishes taking 20 seeds per dish. Six experiment treatments were used: (1) control—seeds non-inoculated; (2) DEMTkZ3A0—seeds inoculated with 1 mL of the DEMTkZ3A0 strain conidia suspension (1.0 *×* 10^5^) prepared as described before (Section 4.5); (3) chitosan—seeds inoculated with 1 mL of chitosan (Sigma-Aldrich, St. Louis, MI, USA) solution (0.05%, *w*/*v*) commercial elicitor; (4) laminarin—seeds inoculated with 1 mL of laminarin from *Laminaria digitata* (Sigma-Aldrich, St. Louis, MI, USA) solution (0.05%, *w*/*v*) commercial elicitor; (5) seeds inoculated with 1 mL of acibenzolar-S-methyl (benzo (1,2,3) thiadiazole-7-carboxylic acid S-methyl ester—BTH) (Acros Organic, New Jersey, NJ, USA) solution (0.05%, *w*/*v*) commercial elicitor; and (6) salicylic acid (SA) (Sigma-Aldrich, Steinheim, Germany) solution (0.05%, *w*/*v*) commercial elicitor. Seeds inoculated with all elicitors were incubated for 5 and 10 days at 20 °C. Then the roots and steams were collected, and their fresh weight was measured (in the unit of g). The roots and stems were frozen in a liquid nitrogen. The experiment was repeated three times.

#### 4.9.2. Study of Root Colonization by *Trichoderma* DEMTkZ3A0 Strain and Wheat Growth Promotion

The ability to colonize by the DEMTkZ3A0 strain of cortical tissue of wheat root was examined on the freshly picked roots after 5 and 10 days of incubation using an Olympus BX53 Upright Microscope equipped with an Olympus XC30 camera and photographed. The promotion of wheat growth by the DEMTkZ3A0 strain was tested on the basis of the fresh weight of the stems and roots in comparison with the weight on the stems and roots in the non-inoculated probe (control) and the trials with commercial elicitors (chitosan, laminarin, BTH, and SA).

After 5 and 10 days of incubation, the concentration of IAA in the roots and steams of wheat inoculated with the DEMTkZ3A0 strain, commercial elicitors, and non-inoculated trial was determined as described before (Section 4.6). IAA concentration was determined in the extraction fluids prepared as described in Section 4.9.3 and was expressed as μg IAA/1 g of fresh weight of stems and roots.

#### 4.9.3. Enzyme Extraction

Roots and stems frozen in liquid nitrogen were grated to the powder in a mortar for 5 min. The enzymes were extracted in eight milliliters (per 1 g of stems or roots) of the modified 50 mM phosphate buffer (pH 7.5), containing 1mM EDTA, 1mM PMSF, and 5% PVPP obtained by shaking at 20 °C, 120 rpm for 15 min. The homogenates were filtered through Miracloth and then centrifuged at 12,000 rpm for 20 min. The entire procedure was carried out at a temperature below 4 °C [148].

#### 4.9.4. Phenylalanine Ammonia Lyase (PAL) Assay

The PAL activity was determined based on the transformation of L-phenylalanine to *trans*-cinammonium acid. The reaction was carried in a borate buffer (pH 8.8) containing 0.03 M l-phenylalanine (Sigma-Aldrich, Steinheim, Germany). The absorbance was measured at 290 nm before the start of incubation and after of 30 min incubation at 30 °C, in quartz cuvettes using the Varian Cary 1E UV–visible Spectrophotometer. The PAL activity was calculated from the linear portion of activity curves and was expressed as nMol trans-cinammonium acid/mg protein released during the hour of incubation based on the difference in absorbance measurements before and after incubation [149,150]. The protein concentration was quantified by the Bradford method [140].

#### 4.9.5. Tyrosine Phenol-Lyase (TAL) Assay

The TAL activity was determined based on the transformation of L-tyrosine to coumaric acid. The reaction was carried out in a borate buffer (pH 8.8) containing 1.9 mM l-tyrosine (Sigma-Aldrich, Steinheim, Germany) solution. Absorbance was measured at 310 nm before the start of incubation and after 30 min of incubation at 30 °C, in quartz cuvettes using the Varian Cary 1E UV–visible Spectrophotometer. The TAL activity was calculated from the linear portion of activity curves and was expressed as nMol of coumaric acid/mg of protein released during the hour of incubation based on the difference in absorbance measurements before and after incubation [150]. The protein concentration was quantified by the Bradford method [140].

#### 4.9.6. Guaiacolic Peroxidase (GPX) Assay

The GPX activity was determined based on the transformation of guaiacol to tetraguaiacol. The reaction was carried in a 100 mM phosphate buffer (pH 6.5) containing 15mM guaiacol (Sigma-Aldrich, St. Louis, MI, USA) and 0.05% H_2_O_2_. The reaction was started by adding of 10 μL of enzyme extract. Absorbance was measured at 470 nm in quartz cuvettes using the Varian Cary 1E UV–visible Spectrophotometer. The GPX activity was calculated from the linear portion of activity curves and was expressed as mMol guaiacol/mg protein released during the one minute [148,151]. The protein concentration was quantified by the Bradford method [140].

#### 4.9.7. Catalase (CAT) Assay

The CAT activity was determined based on the decrease in H_2_O_2_ content in the mixture. The reaction was carried in 50 mM phosphate buffer (pH 7.0) containing 20 mM H_2_O_2_. The reaction was started by adding of 20 μL of enzyme extract. The absorbance was measured at 240 nm in quartz cuvettes using the Varian Cary 1E UV–visible Spectrophotometer. The CAT activity was calculated from the linear portion of activity curves and was expressed as µMol H_2_O_2_/mg protein released during the one minute [148,152]. The protein concentration was quantified by the Bradford method [140].

#### 4.9.8. Chitinase (CHIT) and Glucanase (GLUC) Assay

The CHIT activity was determined in an enzyme extract by the Rodriguez-Kaban et al. [138] method modified by Rössner [139] as well as GLUC activity was determined by the Somogyi [135] and Nelson [136] method modified by Hope and Burns [137], as was described before (Section 4.6). The CHIT activity was calculated from the linear portion of activity curves and was expressed as nMol GLcNAc/mg protein released during the one hour. The GLUC activity was calculated from the linear portion of activity curves and was expressed as nMol glucose/mg protein released during the one hour.

#### 4.9.9. Determination of Total Phenolic Compounds Content in Stems and Roots of Wheat

After 5 and 10 days of incubation, the total phenolic compounds content in the roots and steams of wheat inoculated with the DEMTkZ3A0 strain, commercial elicitors, and non-inoculated trial was determined. Phenols concentration was determined in the extraction fluids prepared as described in Section 4.9.3. The concentration of total phenols was determined by reaction with Folin-Ciocalteau reagent [153]. 0.25 mL of 50% (*v/v*) Folin-Ciocalteau reagent was added to 0.25 mL homogenates and mixed thoroughly. After 3 min, 0.5 mL of 7% Na_2_CO_3_ was added, mixed and incubated for 1 h at 25 °C. The absorbance was measured at 680 nm in quartz cuvettes using the Varian Cary 1E UV–visible Spectrophotometer. The total phenolic compound content was calculated from the linear portion of activity curves and was expressed as μg of ferulic acid (Sigma-Aldrich, Steinheim, Germany)/1g of fresh weight of stems and roots.

### 4.10. Statistical Analysis

All assays were performed in three independent experiments and the data were expressed as means ± SD calculated from these experiments. Standard deviations were determined using Microsoft Excel 2016 (Microsoft Corp., Redmond, Washington, DC, USA). The data were subjected to the one-way analysis of variance (ANOVA) followed by a Tukey’s post hoc test, with the significance evaluated at *p* < 0.05. The principal component analysis (PCA) was performed in the MVSP3.21 package.

## 5. Conclusions and Future Perspective

The results obtained in our studies confirmed that the *Trichoderma–*plant relationship is a very dynamic multi-dependent system, because both partners produce metabolites that interact with each other, and the process of their synthesis is variable over time and depends on biotic and abiotic factors. The specific hormonal regulation and balance is shaped in this relationship. On the one hand, the enzyme regulating the level of plant ethylene (ACC deaminase) and phytohormones (auxin, gibberellin) produced by the *Trichoderma* DEMTkZ3A0 strain can be involved in this regulation. On the other hand, plant’s signal substances induced by the strain colonizing this plant may play a role. After inoculation of wheat seeds with this strain conidia, the colonization of the rhizoplane and root border cells (RBC), an increase in the stem weight and activity of plant resistance markers, and a decrease in the concentration of IAA auxin, were noted.

The strong ability of this strain to inhibit the growth of the phytopathogenic *Fusarium* spp. through the mechanism of mycoparasitism and the very high glucanolytic and chitinolytic activity exhibited by this strain in the presence of pathogenic *F. culmorum* CW are most likely responsible not only for lysis of hyphae and macroconidia of the *Fusarium* spp. but also for the release of plant resistance elicitors.

Thus, the mycoparasitic plant-colonizing strain has great biostimulant and biocontrol potential. This strain can protect plants directly and indirectly by mechanisms of mycoparasitism and by induction of plant resistance pathways, respectively.

Further advanced research on a triple system (*Trichoderma* DEMTkZ3A0 strain–plant–*Fusarium* spp. strains) is needed to confirm these properties in a plant cultivation in different conditions.

At the same time, it is necessary to clarify, using representatives of other *Trichoderma* species, whether the DEMTkZ3A0 strain interaction with RBC and macroconidia and the influence on both plant resistance and hormonal balance, observed in this study but not described in the literature, is typical for the entire genus *Trichoderma*.

## Abbreviations

IAA	indoleacetic acid
GA	gibberellic acid
ACC	1-aminocyclopropane-1-carboxylate
RBCs	root border cells
PAL	phenylalanine ammonia lyase
TAL	tyrosine ammonia lyase
CAT	catalase
GPX	guaiacol peroxidase
GLUC	glucanase
CHIT	chitinase
GAs	gibberellins
ABA	abscisic acid
CWDE	cell wall degrading enzymes
FHB	Fusarium head blight
PGPR	plant growth promoting rhizobacteria
CW	cell wall
ROS	reactive oxygene species
JA	jasmonic acid
PAMP	pathogen-associated molecular pattern
PTI	(PAMP)-tiggered immune signaling pathways
ETI	effector-tiggered immunity
SAR	systemic acquired resistance
MAMPs	microbe-associated molecular patterns
DAMPs	damage-associated molecular patterns
PRRs	plasma membrane-localized receptors
SA	salicylic acid
PR	pathogenesis-related protein
PGPF	plant growth-promoting fungi
SOD	superoxide dismutase
DRMO	deleterious rhizosphere microorganisms
PDA	potato dextrose agar
RB medium	Ryes Byrde medium
ACCD	ACC-deaminase activity
